# Physicochemical Properties of Dentine Subjected to Microabrasive Blasting and Its Influence on Bonding to Self-Adhesive Prosthetic Cement in Shear Bond Strength Test: An In Vitro Study

**DOI:** 10.3390/ma15041476

**Published:** 2022-02-16

**Authors:** Marcin Szerszeń, Julia Higuchi, Barbara Romelczyk-Baishya, Bartłomiej Górski, Witold Łojkowski, Zbigniew Pakieła, Elżbieta Mierzwińska-Nastalska

**Affiliations:** 1 Department of Prosthodontics, Medical University of Warsaw, 02097 Warsaw, Poland; elzbieta.mierzwinska-nastalska@wum.edu.pl; 2 Laboratory of Nanostructures, Institute of High Pressure Physics, Polish Academy of Sciences, 01424 Warsaw, Poland; j.higuchi@labnano.pl (J.H.); w.lojkowski@labnano.pl (W.Ł.); 3 Faculty of Materials Science and Engineering, Warsaw University of Technology, 02507 Warsaw, Poland; barbara.romelczyk-baishya@pw.edu.pl (B.R.-B.); zbigniew.pakiela@pw.edu.pl (Z.P.); 4 Department of Periodontal and Oral Mucosa Diseases, Medical University of Warsaw, 02097 Warsaw, Poland; gorskibartlomiej04@gmail.com

**Keywords:** microabrasive blasting, sandblasting, air-micro-abrasion, microabrasion, shear bond strength, dentine properties, self-adhesive resin cement

## Abstract

The aim of this in vitro study was to assess the influence of microabrasive blasting on the physicochemical properties of dentine and shear bond strength (SBS) of self-adhesive resin cement (Maxcem Elite, Kerr, Orange, CA, USA) bonded to the dentine surface. Ninety cylindrical specimens with exposed dentine of human teeth were prepared and divided into three randomized, parallel sample sets A, B, and C. Groups B and C were subjected to abrasive blasting using a micro-sandblasting device (Microetcher IIa, Danville Materials, Carlsbad, CA, USA) with two gradations of Al_2_O_3_ abrasives (Group B, abrasion with a gradation of 50 μm; group C, abrasion with a gradation of 27 μm). SEM imaging, profilometry, chemical composition analysis, contact angle measurements, surface free energy, and SBS tests were performed. The resulting data were statistically analyzed using the Statistica software (ver. 13.3, Tibco Software Inc., Palo Alto, CA, USA). Microabrasive blasting caused changes in surface topography, structural features, and the connection strength between the dentin surface and self-adhesive prosthetic cement. Air microabrasion through the multifactorial positive reorganization of the treated surface of dentine is recommended as a pretreatment method in fixed prosthodontics adhesive cementation protocols.

## 1. Introduction

Evolution in dental material science and widely understood dental techniques have resulted in changes to or development of new protocols for clinical procedures in the field of prosthodontics. Long-term prosthetic restorations (FDPs), cemented to the surface of mineralized tooth tissues (enamel and dentin), are a prevalent method of tooth reconstruction and rehabilitation of the stomatognathic system, enabling the natural appearance of a smile to be restored so that it is aesthetically and functionally acceptable to patients [1,2,3,4]. Regardless of the choice of method and restorative material, the main goal of dental treatment is to preserve as many patient tissues as possible, hence the evolution from the retention form of prosthetic abutments to preparation procedures based on the use of minimally invasive preparation techniques, defect-oriented preparation techniques, and the global trend known as minimally invasive dentistry (MID) [5,6,7,8].

The development of adhesive methods of cementing prosthetic restorations made it possible to limit the range of preparation, reducing the loss of tooth tissues [9,10]. The possibility of intraoral scanning; implementation of restorations such as veneers, prosthetic onlays, or tabletops; and the use of dental CAD/CAM systems have made it possible to reduce tooth preparation to a necessary minimum and, in some clinical cases, eliminating the need for preparation altogether [11,12,13,14]. Several factors may determine the quality of adhesive processes and thus the long-term clinical success, including the selection of appropriate restorative materials, conditioning the surface of the restoration, pretreatment of the surface of mineralized tooth tissues for adhesive bonding, selection of appropriate cementing materials, and, lastly, a properly performed adhesive cementation protocol [15]. The unfavorable environmental factors enduring in the oral cavity do not show any ability to destroy the current restorative materials. However, despite the well-known phenomena occurring during adhesive cementation processes, the connection of reconstructive material to tooth tissues seems to precisely present a challenge where the appropriate pretreatment of cemented surfaces is crucial [16]. Properly prepared enamel is a tissue with good results in terms of adhesive bonding. Adhesion to dentin is less predictable due to its morphological and histological structure [17,18]. Over the years, numerous different concepts of adhesive bonding to dentin have been developed, sometimes in the form of multi-step, technically challenging procedures. The introduction of single-stage, self-adhesive bonding systems and self-adhesive prosthetic cements to the dental market involved the maximum simplification of clinical procedures and aimed at increasing the strength of the bond between composite materials and dentin. As reported Zecin-Deren et al., Breschi et al., and Hitz et al., among others, at least one of these assumptions was not met: the bond strength of some of these bonding agents with dentin is still inferior to that of existing alternative adhesive methods [19,20,21]. For this reason, many researchers have focused on refining the procedure of chemical conditioning of the dentin surface. It has been proven that the strength of the connection of this type of cement to dentin decreases after its acid etching, which causes loss of the smear layer and damage to the specific scaffold made of collagen fibers, which are crucial to this type of cementation procedure [22,23,24]. Regardless of these reports, cementing materials of this type are often chosen by clinicians because of their simplicity of use [25,26,27,28].

One method of preparing mineralized tissues within the oral cavity is abrasive blasting—more specifically air abrasion—which is in line with the assumptions of the MID [29,30,31]. Dental air abrasion is a relatively simple technique of cavity preparation using an air-abrasive jet, which, by impacting the prepared surface, causes its structural change depending on the adopted processing parameters, such as air pressure or the type or size of abrasive grains used [32,33,34]. In recent years, tools described as abrasive microsandblasters (or microetchers or microblasters), which also involve the kinetic preparation of tooth tissues, have been appearing on the dental market with increasing frequency. However, due to the operating characteristics and construction of this type of tool, the effect of enamel and dentin preparation is different than in the case of traditional abrasive sandblasters [35,36]. Abrasive microsandblasting machines are handle-shaped devices with a bent nozzle tip and an abrasive powder container integrated with the body. In the case of microabrasive blasting, the Venturi principle is responsible for the formation of an abrasive air jet. This effect causes the abrasive powder from the tank to be transported by creating a vacuum within the suction tube, which is connected at the end of the handle to a pressure conduit reaching the narrowed rear part of the nozzle, from which the air-abrasive stream emerges. Microsandblasting machines are most often used as handpieces installed on a turbine sleeve (e.g., MicroEtcher CD, Danville Materials, Carlsbad, CA, USA), as handles connected to a source of compressed air derived from the control unit, or the compressor of a dental unit (e.g., Airsonic Mini Sandblaster, Hager Werken, Duisburg, Germany; MicroEtcher IIa, Danville Materials, Carlsbad CA, USA; MicroBlaster, Bio-Art, Sao Carlos, SP, Brasil; Dento-Prep, Ronvig, Daugard, Denmark). The first type of connection enables the operation of abrasive microsandblasters with the foot controller of the unit. The second type requires a different handle structure, which must be equipped with a trigger button. This switch is most often located on the side surface of the working tip to enable operation with the thumb. Such sandblasters do not allow the adjustment of parameters such as pressure or the amount of abrasive emerging from the nozzle. Theoretically, it is possible to change the geometry or the macrostructure of a tooth with the use of abrasive microsandblasters, but that would constitute an intensely laborious and time-consuming process due to the limited effectiveness of these devices and the use of nozzle orifices wider than in air-abrasion units, creating a wider abrasive jet [37]. Microstructural changes in the form of increased roughness and development of the adhesive surface are the leading reasons given by producers for choosing this type of device, so they are widely used in orthodontic bracket placement to enamel [38,39,40,41,42].

We had not encountered research on the influence of sandblasting with the use of abrasive microsandblasting devices on dentine properties such as surface microgeometry, the chemical composition of the surface layer, contact angle, free surface energy of the sound dentine/dentine smear layer, and improvement in the quality of cementing adhesive procedures. Therefore, the aim of this study was to evaluate the physicochemical properties of dentin subjected to microabrasive blasting and its influence on the bond with the self-adhesive prosthetic cement. Our null hypothesis was that no differences exist in the physicochemical properties and shear bond strength between microabraded and nonmicroabraded dentine.

## 2. Materials and Methods

### 2.1. Sample Preparation and Study Design

**Figure 1:**
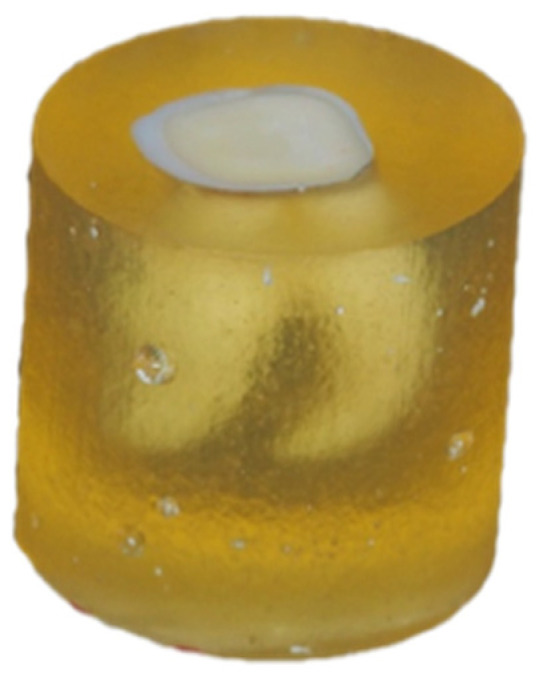
Prepared tooth embedded in epoxy resin after polishing with waterproof sandpaper and rinsing.

We used 90 human third molars, partially retained or fully erupted, free of caries and without dental fillings, removed for orthodontic or surgical indications. Local protocols, reviewed and approved by the Ethics Committee of the Medical University of Warsaw, were followed. Each tooth was prepared with a diamond bur (6850.314.012, Gebr. Brasseler GmbH & Co. KG., Lemgo, Germany) at approximately 1/3 of the height of the clinical crown. The adopted protocol aimed to expose the largest possible surface of the dentin. Teeth prepared in this manner were embedded in epoxy resin and polished with P120-graded waterproof sandpaper, then P600-graded sandpaper (according to the FEPA; Figure 1). The material was divided into three randomized, parallel sets of samples A, B, and C by means of an online research randomizer (www.randomizer.org (accessed on 8 December 2020)). Groups B and C underwent abrasive blasting using a microsandblasting device (Microetcher IIa, Danville Materials, Carlsbad, CA, USA), with the use of two gradations of Al_2_O_3_ alumina abrasives (Group B, abrasive with a gradation of 50 μm; group C, abrasive with a gradation of 27 μm). Settings for the distance and angle of inclination of the microsandblaster nozzle in relation to the surface of the sandblasted tooth, as well as the air pressure supplied directly from the compressor, were identical in all instances: distance: 4–5 mm, angle: 60, and pressure: 70 ± 2 psi. The preparation method involved a smooth movement of the nozzle of the abrasive microsandblaster from left to right for 15 s. Group A was the control group. After preparation, samples were thoroughly rinsed off for 10 s using a dental unit air–water syringe and subsequently stored in distilled water.

### 2.2. Scanning Electron Microscope (SEM) and Profilometry

Five samples from each of groups A, B, and C were analyzed for the microgeometry of the dentine surface subjected to air microabrasion. Firstly, the samples were imaged using a scanning electron microscope (Ultra Plus, Zeiss, Germany), producing 60 microscopic images (20 for each group). This study was used to provide information on possible changes in the characteristics of the dentin surface. The second part of the study involved imaging the topography of the dentin surface layer of the samples, recorded with a digital microscope (VHX-7000, Keyence, Belgium). The images obtained were exported in the form of three-dimensional reconstructions of the surface layer and subjected to profilometric analysis. Data obtained from the analysis were recorded in the form of Ra and Rz parameters and were used to provide information on any changes in the roughness characteristics of the dentine subjected to abrasive blasting. The Ra parameter is the arithmetic mean of the filtered roughness profile, determined on the basis of deviations from the centerline within the assessment length. The Rz parameter is the greatest height of the roughness profile: sum of the height of the highest profile peak and the depth of the deepest profile valley, relative to the mean line, within an assessment length. Each sample was subjected to a three-fold linear roughness analysis, obtaining a total of 3 data series of 15 components. 

### 2.3. Chemical Composition Analysis

For the chemical composition analysis of the dentine subjected to the air microabrasion technique, scanning electron microscopy with X-ray energy dispersive spectroscopy (EDX) was used. For this part of the study, 3 samples from each group were qualified, and the analyzed sites were randomly selected within the dentin, obtaining at least three images for each of the tested samples. We used the option of elemental mapping, highlighting the imaging of chemical elements: aluminum, calcium, and phosphorus. Observations of this type were performed to provide information on possible elemental changes within the outer layer of dentine subjected to abrasive air microabrasion.

### 2.4. Static Contact Angle and Surface Free Energy (SFE) Tests

The static contact angle (CA) and the surface free energy (SFE) were measured for five samples from each group (A, B, and C). The test was performed using a goniometer (DSA 25B, Krüss GmbH, Hamburg, Germany). The contact angles of samples in air and controlled environment (temperature, 22 °C; humidity, 40%) were measured for three types of test liquids (1-bromonaphthalene, diiodomethane, and deionized water) applied to the prepared dentin. Samples were kept in environmental conditions of 22 °C and 40% humidity for 24 h before the measurement to minimize the variability in materials’ surface properties. Digital measurements were performed with a 10 μL droplet using a syringe equipped with a needle placed every 10 s (10 droplets per sample). The surface free energy was calculated based on the results of the contact angles using the distilled water calculation method according to the equation proposed by Robeson [43]:

SFE = 74.5 − 0.372x − 0.00181x^2^ (x = contact angle of distilled water) (1)

and using 3 test liquids (Owens−Wendt−Rabel−Kaelble (O-W-R-K) method). This procedure was applied to provide information on possible changes in the contact angles and the surface free energy of the dentine subjected to the abrasive blasting method in the form of air microabrasion.

### 2.5. Shear Bond Strength (SBS) Test

**Figure 2:**
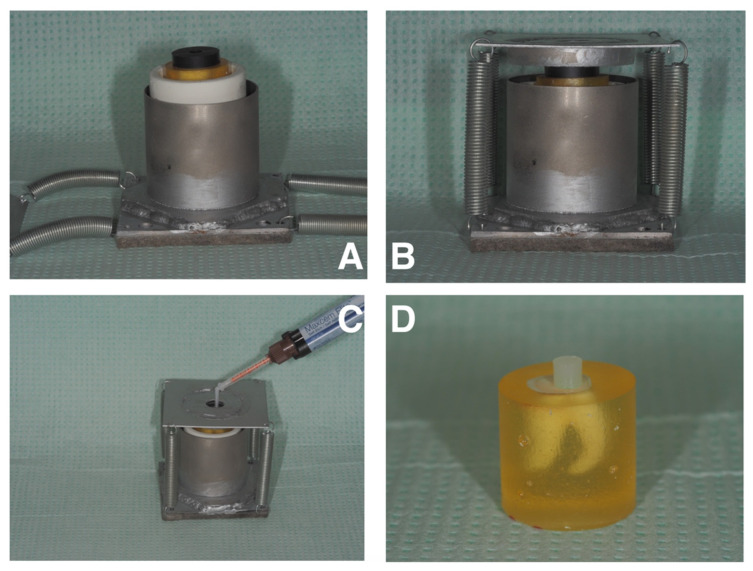
Sample preparation for shear bond strength tests. (**A**) Specimen is placed in the BSM1 tool, and the rubber cylinder with opening is placed on the test surface of the sample; (**B**) pressing the rubber cylinder against the sample surface by a platform engaged with tension springs; (**C**) applying cement to the opening of the rubber cylinder; (**D**) sample after being released from the BSM1 tool.

Static shear tests were performed to measure the strength of the connection between dentine subjected to microabrasive blasting and self-adhesive composite cement. We used 75 samples (25 samples each from groups A, B, and C) for this section of the study. In order to carry out shear bond strength tests on the prepared dentin surfaces, analogs of prosthetic restorations in the form of cylinders of the self-adhesive Maxcem Elite composite material (Kerr, Orange, CA, USA) 4 mm in diameter and 4 ± 1 mm in height were cemented by means of a device (BSM1) designed specifically for this research, enabling repeatable sample preparation (Figure 2). After storing in distilled water at 37 ± 1 °C for 24 h, 75 shear bond strength tests (SBSs) were performed using a Zwick/Roell Z005 universal testing machine (ZwickRoell GmbH, Ulm, Germany) equipped with a 1 kN load cell. The crosshead velocity was 1 mm/min for each test. The aim of the test was to provide information on the strength needed to debond the composite cement analogs from the dentin surface for 3 groups of samples A, B, and C. The base of force registered during the tests and specimen’s cross-section stresses for each sample were calculated. All SBS-tested specimens were photographed using a digital microscope (DTX 50, Levenhuk, Tampa, FL, USA) and native software at 20× magnification to determine the adhesive remnant index (ARI). This scale ranges from 0 to 3. A score of 0 indicates no adhesive remaining on the tooth in the bonding area; 1 indicates less than half of the adhesive remaining on the tooth; 2 indicates more than half of the adhesive remaining on the tooth; 3 indicates all adhesive remaining on the tooth [44].

### 2.6. Statistical Analysis

Data obtained from tests in which quantitative results were obtained were statistically analyzed using Statistica software (ver. 13.3, Tibco Software Inc., Palo Alto, CA, USA). Descriptive statistics including mean and standard deviation were performed. The normal distribution of data was verified using Shapiro–Wilk tests. One-way ANOVA or Kruskal–Wallis tests and post hoc tests were performed in groups with statistically significant differences. The level of significance for tests was set at *p* < 0.05.

## 3. Results

**Figure 3:**
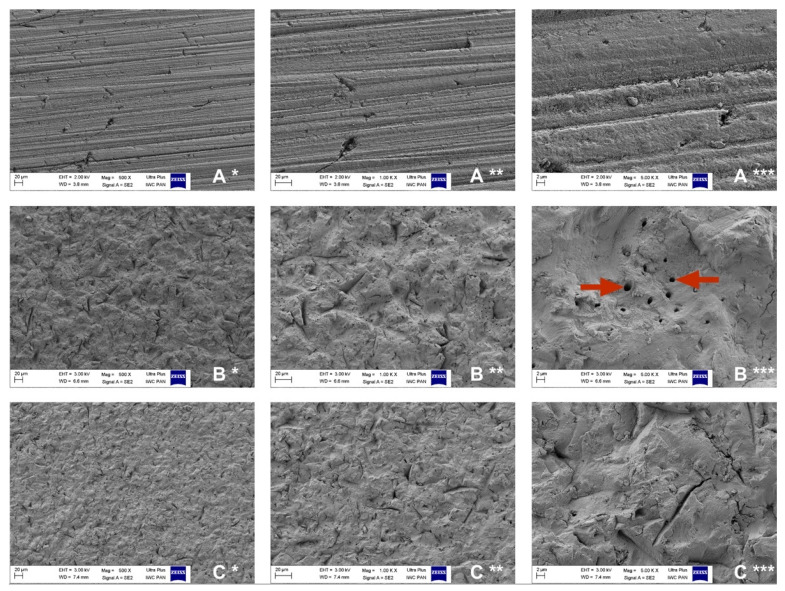
Scanning electron microscopy (SEM) of dentine samples in control group (**A**), 50 μm microabrasive blasting group (**B**), 27 μm microabrasive blasting (**C**), at 500× magnification (*), 1000× magnification (**), and 5000× magnification (***). Red arrows—exposed dentinal tubule orifices.

Based on the results of the conducted research, we concluded that abrasive blasting in the form of air microabrasion significantly changes the characteristics of dentin, regardless of the gradation of alumina abrasive used. The obtained microscopic images showed the modified surface of the dentin smear layer in the form of folds, numerous depressions, and grooves of irregular shapes and sizes in both research groups compared to the surface of the control group, which was characterized by relatively homogeneous surfaces. In the group treated with the 50 μm Al_2_O_3_ abrasive, smear-free areas with numerous 0.3–1.5 μm openings corresponding to exposed dentinal tubule orifices were noted (Figure 3).

**Figure 4:**
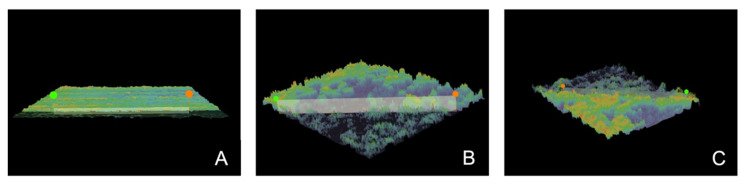
Representative 3D projections of dentine surface layers obtained by a VHX-7000 digital microscope in the group without microabrasive blasting (**A**) and prepared by means of a microsandblaster with the usage of 50 (**B**) and 27 μm (**C**) Al_2_O_3_.

The results show that dentine subjected to microabrasive sandblasting, regardless of the size of the abrasive grains, was characterized by roughness parameters Ra and Rz many times greater than those of dentine samples from the control group (Figure 4). The profilometric study showed that the group subjected to air microabrasion with the use of alumina abrasives with a gradation of 27 μm had the highest roughness values according to the Ra and Rz parameters. Differences between the groups in terms of Ra and Rz parameters were statistically significant (ANOVA *p* < 0.00000); therefore, post hoc Games–Howell testing was also conducted, as presented in Table 1. The descriptive statistics of profilometry data, ANOVA and post hoc tests are presented in Table 1.

**Figure 5:**
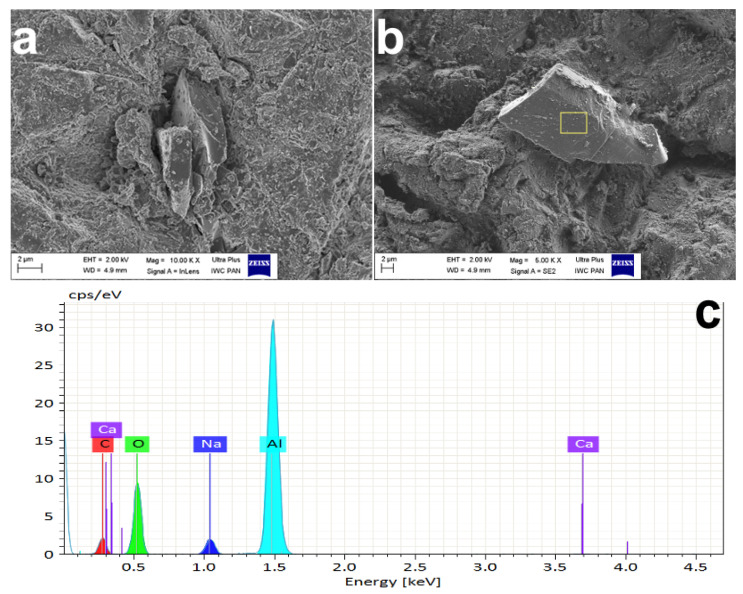
(**a**,**b**) Aluminum oxide grains embedded in dentine/smear layer; (**c**) graphical representation of the elements in the outer layer of the area of sample B marked on the illustration.

**Figure 6:**
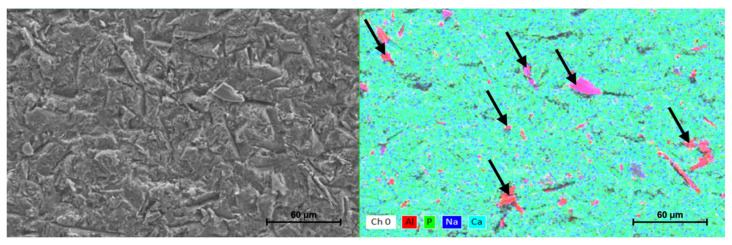
EDS analysis with mapping of the surface of the dentin sample. The arrows indicate aluminum clusters. Scanning electron microscope images, 1000× magnification.

Based on the conducted research, we found that microabrasive blasting using both 27 and 50 µm alumina left abrasive particles of various sizes and shapes on the dentin tissue/smear layer, which settled or stuck to its surface (Figure 5). The elemental mapping showed the presence of aluminum clusters on all imaged dentin surfaces of groups B and C. No pattern regarding the distribution and number of abrasive particles in the visualized areas was observed (Figure 6).

The analysis of the CA of the dentin surface showed that microabrasive blasting changed the dentin wettability independent of the sand grain gradation. Descriptive statistics of the contact angles and SFE are presented in Table 2. Differences between tested groups, regardless of the type of solution used to determine the static contact angle, were statistically significant (ANOVA, *p* < 0.000001), but the largest differences were observed between the control group and the test group C; the post hoc Scheffe testing results are shown in Table 2. In the assessment of surface free energy, both for the method based on calculations in accordance with the formula for the contact angles with deionized water (proposed by Roberson) and in the O-W-R-K method, statistically significant differences were obtained between groups (ANOVA, *p* < 0.0001). The results showed an increase in the surface energy of the dentine blasted with a microetcher, regardless of the gradation of the alumina abrasive used and the differences were statistically significant; the post hoc Scheffe testing results are shown in Table 2.

**Figure 7:**
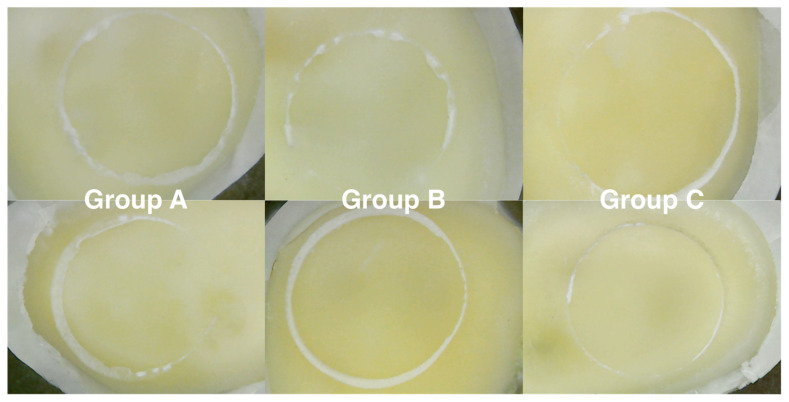
Sample images of specimens after SBS testing in different groups (20× magnification).

Shear bond strength test results showed that microabrasive blasting, using both 27 and 50 μm graded alumina abrasives, increased the force needed to debond the cylinder of self-adhesive composite cement from the dentin surface compared to the force needed for samples not subjected to this type of preparation. The descriptive statistics of SBS tests data are presented in Table 3. The highest median shear strength was obtained for the group subjected to microabrasive Al_2_O_3_ sandblasting with a particle size of 27 µm (group C): 6.25 MPa. The Kruskal–Wallis test results showed that there was a significant difference between groups (*p* < 0.00000). The difference between experimental groups was statistically significant compared to the results of the control group (post hoc Dunn tests). There was no statistically significant differences between experimental groups B and C. Figure 7 depicts sample photographs of SBS-tested specimens. The fracture pattern was adhesive in every specimen tested (ARI score of zero).

## 4. Discussion

In the present study, we assessed the microgeometry, profilometry, chemical composition, contact angle, and surface free energy of the dentine surface subjected to micro abrasive blasting. In addition, the SBS to self-adhesive resin cement of the subjected dentine was evaluated. The results rejected our null hypothesis because differences between groups were noted in all performed investigations.

The formation of a smear layer after the preparation of dentin with dental burs is a commonly known phenomenon [45,46]. In the present study, we chose to polish the dentin surface of all tested samples to unify the surface of the smear layer and to eliminate the influence of dentin shaping on further research procedures through the preparation of the tested teeth with a bur. The selection of waterproof abrasive paper for polishing depended on the gradation of the grain size and was intended to be comparable to the final tooth preparation in clinical conditions. P600 abrasive paper, also used by other researchers in similar studies, has abrasive particles with a gradation of approx. 20–25 µm, which is comparable to the grit of a dental superfine diamond bur (dental diamond burs with yellow marking) for the final smoothing of the tooth preparation [47,48,49]. Preparation of dentin with the use of the microabrasive blasting method resulted in the formation of a significantly different surface of the smear layer, and in the case of using the Al_2_O_3_ abrasive with a grain gradation of 50 µm, it was completely removed in numerous areas, revealing the orifices of the dentinal tubules. Microabrasive blasting with 27 µm alumina produced heterogeneous surface microgeometry, but only in terms of the thickness of the smear layer; similar dentin illustrations obtained from electron microscope scans were obtained by Raczyńska et al. [50]. A similar image of dentin, or de facto of a smear layer of dentin, was also obtained by Chinelatti et al. [32] and Mujdeci et al. [51], among others. However, these researchers employed the abrasive blasting method in the form of traditional air abrasion, and not, as was the case in the present study, with the use of an abrasive microsandblasting device.

Regardless of the characteristics of the abrasion method, the final image showed that the outer surface of the dentin subjected to abrasive blasting is also influenced by factors such as the distance of the nozzle from the surface of the prepared tooth tissue, the working pressure of the sandblasting tool, the preparation time, and the container filling level [31,33,52,53]. Lima et al., in systematic review and meta-analysis, after interpreting 32 studies on classical air abrasion, concluded that airborne-particle abrasion (APA) has no negative effect on bonding to dentine, but a positive effect can only be achieved under certain abrasive sandblasting conditions. To improve the bond strength, they suggested using particles larger than 30 μm and an air pressure greater than 5 bar [54]. On the other hand, Ouchi et al. found that after abrasive blasting of the surface with alumina particles, the dentin bond strength of universal adhesives in self-etching mode decreased. They theorized that the adverse effect was caused by the smear layer’s compaction after alumina blasting, which could prevent the adhesive resin from penetrating [55]. Nevertheless, the effect of APA on the formation, compaction, or removal of the dentin smear layer is still the subject of many studies, which have often produced contradictory conclusions [47,56,57,58,59,60].

Contrary to the results of the present study, Manhart et al. [61] did not find a difference in the surface morphology after using identical sand gradations (27 and 50 µm); however, they employed nearly twice the working pressure (160 psi) during the air abrasion of dentin. Banerjee et al. [62] also proved that the level of filling of the abrasive reservoir in abrasive sandblasters impacts on the powder flow rate (PFR) indicator. We also noticed such a tendency with the use of a microsandblaster (Microetcher IIa), but it was not the subject of the study and requires further research.

Due to the removal of a portion of the dentin smear layer during abrasive micro-blasting, changes in surface roughness also occurred. In this study, in order to evaluate the characteristics of changes in surface microgeometry, parameters Ra and Rz were calculated, which are the most frequently used roughness parameters for this type of study [63,64,65,66]. Air microabrasion with grain gradations of both 27 and 50 µm alumina produced a surface much rougher than the relatively flat one achieved after polishing only. Notably, the group subjected to the lower-graded abrasive showed higher mean roughness values both in the arithmetic mean deviation from the mean line (Ra) and in the highest roughness height according to the 10 highest profiles measured (Rz). The possible explanation of this result is that Al_2_O_3_ particles with a 50 µm gradation, having a larger mass, were characterized by a much higher kinetic energy when impacting the tooth surface, causing not only defects in the smear layer but also reducing its thickness and exposing the surface of the dentin. On the contrary, the lower-gradation abrasive produced deeper defects on the surface but only in certain areas of the smear layer; other areas were almost intact, generating relatively deep craters with a significant difference in level. No analogous or similar studies assessing the roughness of the dentin surface/smear layer after microabrasive blasting are available in the literature for comparison of the results of the Ra and Rz parameters. Most of the available literature on this subject refers to the dentin roughness depending on the use of various types of dental burs, etching with various acids or ultrasound cleaning, most often with the complete removal of the smear layer [67,68,69].

The existing types of powders are assigned to a specific type of treatment in the form of sandblasting. Most of the materials of this type available on the market are recommended for prophylactic sandblasting (sodium bicarbonate, glycine, and calcium carbonate), and these are usually materials with a lower hardness than tooth tissue (Mohs hardness: enamel, approx. 5; dentin, approx. 3–4) [70,71,72]. The aluminum oxide used in this study is characterized by the highest hardness among all dental abrasives; it is meant for use in air abrasion and microabrasion treatments and, as a rule, modifies it during kinetic impact on the tooth structure.

Surface topography is well-known to have a significant influence on the interaction between bacteria and substrates, not only in connection with human health disorders but also in a variety of other fields [73]. Despite numerous benefits, intraoral sandblasting, both in the form of traditional air abrasion and microabrasive blasting, has some disadvantages. Changes in the roughness or surface free energy of tooth tissues can be interpreted as advantageous in terms of bonding with dental cements, but the iatrogenic effect of the air-abrasive jet may increase the possibility of bacterial biofilm formation. The surface topography determines the level of microbial adherence. Additionally, if bacteria colonizes an irregular surface, they will proliferate and develop a biofilm, which is harder to eradicate by standard methods [74,75]. Conversely, some authors suggested that the free surface energy has a greater influence on bacterial adherence than surface roughness [76]. If the operator fails to properly protect the tissues that should remain intact during the microabrasive sandblasting procedure, the surface will be predisposed to the formation of bacterial plaque, especially considering the natural presence of many strains of bacteria in planktonic form in human saliva [77,78]. Taking the above into account, additional tissue-protection elements in the form of a rubber dam or liquid dam are strongly recommended.

According to some authors, not only the microgeometry but also the chemical content of the smear layer and dentin may change under the influence of air abrasion [51,56]. Examination of the elemental composition of the outer layer of samples subjected to air microabrasion, carried out in this study, confirmed that aluminum oxide clusters visualized using the element mapping method are deposited in the smear layer and surface, regardless of the abrasive gradation. It has also been documented that it is not possible to remove these particles with the use of an air–water stream of a three-function syringe in the dental unit, which has been discussed by other authors [57,79,80].

Along with the increases in roughness and specific surface area, changes in the wettability of the prepared tissue may occur because of the process. According to Bieliński et al., wettability is a physical property that determines the basic functions of materials, e.g., adhesion [81]. The clear influence of roughness with characteristic wettability properties, such as contact angle or surface free energy, and its influence on the ability of dentin bonding to dental adhesive cements, have not been clearly defined. Manhart et al. [61] and Mujdeci et al. [51] reported that increased roughness also increases the possibility of wetting enamel and dentin with bonding systems. In contrast to their research, Al-Omari et al. [67] did not observe an increased wettability of the tooth tissue surface with distilled water with increasing roughness. Inoue et al. [82] studied the roughness and surface free energy after applying self-etching bonding systems to dentin, observing an increase in energy with a reduction in roughness for two of the three tested systems. However, the above-mentioned authors did not test the wettability after dentine preparation using microabrasive blasting methods. Our study of the contact angle was performed with the use of three types of liquid. This procedure allowed us to not only to visualize the behavior of liquid droplets with different characteristics (polar and nonpolar) on the dentin surface, but also to calculate the total surface free energy using both the O-W-R-K method and the formula proposed by Roberson. This provided information on the differences resulting from the mathematical transformations, and not from the differences in the examined empirical properties [43,56,65,83]. The inclination angles of the tangent to the droplet surface at the point of its contact with the substrate, noted in this study, proved the relationship between microroughness and wettability. The samples subjected to air microabrasion with the use of 27 µm alumina, characterized by the highest roughness values, created the smallest contact angles with all test liquids.

Since dentin is a highly heterogeneous structure in which, unlike enamel, a significant organic component eliminates the positive aspects of phosphoric acid etching, the formation of an adhesive bond also requires different protocols. While etching of enamel almost doubles surface energy, applying the same acid to dentin tissue has the opposite effect [84]. According to Pospiech [85], the value of the dentin surface energy, estimated at 42–45 mN/m, after etching, decreases to 27–30 mN/m. The values obtained in the present research for dentine subjected to abrasive sandblasting were approximately 55 and 45 mN/m for the groups subjected to 27 and 50 µm alumina, respectively.

The influence of air abrasion on the values of shear tests has been widely described in the literature in the last few decades. Despite numerous reports on this subject, it is still not possible to establish a consensus due to the occasional contradictory reports regarding the positive or negative influence of abrasive blasting on the possibility of bonding with composite materials even with similar test methodologies. In Souza-Zaroni et al. [86,87], Mujdeci et al. [51], and D’Amario et al. [88], the authors reported a positive effect of air abrasion methods on adhesive bonding. On the contrary, among others, Borsatto et al. [89] and Huang et al. [38] did not find a statistically significant positive effect of this type of pretreatment, and Manhart et al. [61] obtained results comparable to the use of orthophosphoric acid etching, which is currently the gold standard of chemical pretreatment methods of mineralized tooth tissues prior to adhesive bonding.

In most of the available studies on the effect of APA on SBS, self-adhesive prosthetic cements were not used; the classic adhesive methods in the form of the etch-and-rinse approach were used. These methods are based on etching, applying a bonding system and subsequent fixing of the composite material. The operating module of these two protocols is completely different. In the case of self-etching, self-adhesive resin cements (consisting of three main components: conventional mono-, di-, and/or multimethacrylate monomers; acid monomers; and fillers), the dissolution of the smear layer, is possible due to the content of acid monomers acting in the first stages when applied on mineralized tooth tissues [90]. Depending on the specific pH of the material, the possibility of dissolving the smear layer may or may not reach the dentinal tubules. The acidic monomer content in these materials must be balanced to achieve an acceptable degree of self-etching and bonding to dentin and enamel while avoiding excessive hydrophilicity in the final polymer [91]. Another difference between this type of material and standard treatment (in the form of etching with phosphoric acid) is the status of etched minerals. The etching pattern of strong self-etching adhesives (pH ≤ 1) mimics that of phosphoric acid; however, the dissolved mineral content is not washed away. As a result, the dentin connection might be weakened by dissolved mineral remains that must be incorporated into the resin layer. The mild (pH ~2) and ultra-mild self-etching adhesives (pH ≥ 2.5), conversely, demineralize the dentin surface just superficially; if the smear layer is dense, they cannot penetrate it [59,92]. Lastly, as shown by Breschi et al. self-etch adhesives often infiltrate no further than the smear layer and smear plugs, revealing a more homogenous morphology that is devoid of long resin tags. Micromechanical interlocking is still considered the primary mechanism of adhesion to enamel and dentine. This may be one of the main reasons for the low SBS test averages compared to those of etch-and-rinse adhesive systems since they infiltrate dentin tubules funneled by the etching agent and create deep resin tags [20,93].

The shear bond strength tests conducted in the current study proved that the additional procedure, in the form of microabrasive blasting, resulted in a significant increase in the force needed to debond the composite material from the dentin surface, regardless of the size of the abrasive particles used. The resulting mean values for both groups treated with alumina were more than twice as high as in the control group. The median forces needed to separate the cement from the dentin in the study were 6.12 MPa for the group in which 50 µm Al_2_O_3_ was used; 6.25 MPa for the group subjected to air microabrasion with 27 µm Al_2_O_3_, and 2.91 for the control group in which the smear layer on the dentin surface was left intact. We found only one study that used the same self-adhesive cement in the SBS test to dentine in which, in contrast to our study, bovine teeth were used [94]. Mean shear bond strength to dentine levels obtained both in the above-mentioned and the present study differed significantly from the value of 21 MPa quoted by the manufacturer [95].

Notably, the presence of alumina grains embedded in the dentin surface did not adversely affect the results of the SBS tests. The multi-shaped, sharp-contoured grains containing significant amounts of aluminum most likely acted as additional anchorages or were possibly absorbed into the cement resin mass during the first stages of dissolving the inorganic compounds of the smear layer. Alumina particles themselves were used in research as refiners of some resins, to give them better mechanical properties. It has been proven, inter alia, that the weight content oscillating up to about 20% improves these properties in the case of Al_2_O_3_/epoxy resin or as a biocidal modification of the surface of endosseous implants. Such surface modification reduced adhesion of bacteria to Al_2_O_3_-blasted surface [96,97,98]. Considering the above, the incorporation of alumina particles into the content of already set prosthetic cement does not appear to be a drawback, nevertheless, whether it creates mechanical or biological alterations in the material itself is beyond the scope of this study and requires additional investigation.

The existing imperfections of laboratory tests in relation to in vivo conditions do not change the opinion of many authors that the results can be adequately related to the clinical situation, and that they can be successfully used to assess the physico-chemical and mechanical parameters of materials. It seems that such tests are of particular importance in cases where it is not possible to perform analogous examinations in an intraoral environment [99,100,101].

Our research has some plausible limitations. The main disadvantage is that there are imperfections in laboratory tests in relation to in vivo conditions. This does not change the opinion of many authors that the results can be adequately related to the clinical situation and that they can be successfully used to assess the physico-chemical and mechanical parameters of materials. Such tests are of particular importance in cases where it is not possible to perform analogous examinations in an intraoral environment [84]. Secondly, the thickness of the smear layer present in our study may be the cause of the low SBS scores of the groups. Despite the use of the polishing protocol, which was also employed in other studies, the thickness of the resulting smear layer was not measured, and could have been higher than after standard dental preparation. In such a case, the use of self-adhesive cement might not have allowed the smear layer to completely dissolve due to the insufficient activity of the acidic monomers contained in the cement we used. It is well-known that single-step, self-etching cements infiltrate no further than the smear layer and smear plugs; in our studies, in certain areas of dentin, resin could only infiltrate the smear layer without penetrating the dentinal tubules at all. Thirdly, due to the significant variability in dentin, adhesion may be significantly influenced by parameters such as the patient’s age or the degree of tissue mineralization, as well as the depth of preparation or the distance from the dentin-enamel junction (DEJ). The dentinal tubules, depending on the distance between the DEJ and the pulp chamber, have different diameters and courses, which may be the reason for high values of the standard deviation in our SBS test. Finally, no complex thermocycling was employed in this study; instead, a 24 h water bath was used. The adopted methodology is common in this type of research, but that does not change the fact that cyclic temperature changes affects the adhesive bond, which allows for a more accurate interpretation of the influence of laboratory investigation results on long-term intraoral circumstances.

## 5. Conclusions

Despite the limitations of an in vitro study, we concluded that microabrasive blasting causes visible changes in terms of surface topography, structural features, and the connection strength between the dentin surface and self-adhesive cement. Air microabrasion increases the roughness parameters and contributes to the enlargement in the adhesion area of the cementing material to the dentine. Moreover, abrasive alumina particles cause changes in the chemical composition of the top layer of dentin. Abrasive blasting in the form of air microabrasion increases the wettability and surface free energy of dentine. In addition, we proved that the microabrasive blasting method, with the use of 27 or 50 µm alumina, through the multifactorial positive reorganization of the treated surface, increases the strength of the bond between dentine and self-adhesive prosthetic cement. Microabrasive blasting can be a promising, fast, cost-effective, and patient-friendly additional technique used during the preparation of teeth. In our opinion, the procedure can be recommended in the protocol of creating fixed long-term prosthodontic restorations, in particular those cemented with self-adhesive composite cements.

## Figures and Tables

**Table 1:** Descriptive statistics of the different groups for Ra and Rz parameters (μm) as well as the F and *p*-value of one-way analysis of variance.

	Mean (SD)		Min		Median		Max		F		*p*	
	Ra	Rz	Ra	Rz	Ra	Rz	Ra	Rz	Ra	Rz	Ra	Rz
Group A	0.40 ^bc^ (0.06)	2.67 ^bc^ (0.17)	0.28	2.43	0.42	2.66	0.47	2.99	6964.65	11614.09	0,00000 *	0,00000 *
Group B	12.63 ^ac^ (1.16)	84.08 ^ac^ (2.22)	10.22	81.41	12.73	84.20	14.03	88,20				
Group C	16.97 ^ab^ (0.45)	108.71 ^ab^ (3.61)	15.99	100	16.98	109	17.57	113				

Group A, control group; SD: standard deviation; Min: minimal value; Max: maximal value; Games–Howell post hoc: ^bc^ statistically significant in comparison to group B and C, ^ac^ statistically significant in comparison to group A and C, ^ab^ statistically significant in comparison to group A and B; * *p*-value < 0.05 is statistically significant.

**Table 2:** Descriptive statistics of the different groups for contact angle (°) and SFE (mN/m) as well as the F and *p*-value of one-way analysis of variance test.

			Group A	Group B	Group C	F	*p*
Contact angle (^o^)	Deionized water	Mean (SD)	92.28 ^bc^ (1.35)	83.65 ^ac^ (4.37)	41.93 ^ab^ (6.26)	217.06	0.00000 *
		Min/Max	90.20/93.30	77.0/89.40	31.0/48.80		
	1-bromonaphtalene	Mean (SD)	50.28 ^bc^ (2.96)	35.75 ^ac^ (3.93)	22.47 ^ab^ (2.87)	107.42	0.00000 *
		Min/Max	45.40/53.30	31.90/41.50	19.40/27.10		
	Diiodomethane	Mean (SD)	60.54 ^bc^ (3.79)	45.40 ^ac^ (3.93)	36.03 ^ab^ (3.83)	61.74	0.00000 *
		Min/Max	55.35/65.40	38.50/49.40	32.20/41.0		
SFE (according to Roberson method) (mN/m)		Mean (SD)	24.75 ^bc^ (0.87)	30.69 ^ac^ (2.68)	55.66 ^ab^ (2.95)	242.41	0.00000 *
		Min/Max	23.96/26.22	26.78/35.12	52.03/61.23		
SFE (according to O-W-R-K method) (mN/m)		Mean (SD)	22.57 ^bc^ (5.98)	33.42 ^ac^ (5.77)	46.97 ^ab^ (5.73)	22.01	0.00009 *
		Min/Max	17.06/31.40	28.11/41.20	39.23/54.12		

Group A, control group; SD: standard deviation; Min: minimal value; Max: maximal value; Scheffe post hoc: ^bc^ statistically significant in comparison to group B and C, ^ac^ statistically significant in comparison to group A and C, ^ab^ statistically significant in comparison to group A and B; * *p*-value < 0.05 is statistically significant.

**Table 3:** Descriptive statistics of the different groups for SBS tests (in MPa) as well as the F and *p*-value of Kruskal–Wallis test.

	Mean (SD)	Median	Min	Max	F	*p*
Group A	2.892 ^bc^ (1.68)	2.911	0.811	9.10	35.18	0.00000 *
Group B	6.736 ^a^ (2.79)	6.121	2.812	13.17		
Group C	6.677 ^a^ (3.41)	6.250	2.461	16.89		

Group A, control group; SD: standard deviation; Min: minimal value; Max: maximal value; Dunn post hoc: ^bc^ statistically significant in comparison to group B and C, ^a^ statistically significant in comparison to group A; * *p*-value < 0.05 is statistically significant.

## Data Availability

The data presented in this study are available on request from the corresponding author.
